# Successful Nonoperative Management of High-Grade Blunt Renal Injuries

**DOI:** 10.1155/2016/3568076

**Published:** 2016-11-27

**Authors:** Allison M. May, Oussama Darwish, Brian Dang, John J. Monda, Prajakta Adsul, Johar Syed, Sameer A. Siddiqui

**Affiliations:** Department of Surgery, Urology, Saint Louis University, 3635 Vista Ave, St. Louis, MO 63110, USA

## Abstract

Current management of high-grade blunt renal trauma favors a nonoperative approach when possible. We performed a retrospective study of high grade blunt renal injuries at our level I trauma center to determine the indications and success of nonoperative management (NOM). 47 patients with blunt grade IV or V injuries were identified between October 2004 and December 2013. Immediate operative patients (IO) were compared to nonoperatively managed (NOM). Of the 47 patients, 3 (6.4%) were IO and 44 (95.6%) NOM. IO patients had a higher heart rate on admission, 133 versus 100 in NOM (*P* = 0.01). IO patients had a higher rate of injury to the renal vein or artery (100%) compared to NOM group (18%) (*P* = 0.01). NOM failed in 3 of 44 patients (6.8%). Two required nonemergent nephrectomy and one required emergent exploration resulting in nephrectomy. Six NOM patients had kidney-related complications (13.6%). The renal salvage rate for the entire cohort was 87.2% and 93.2% for NOM. Nonoperative management for hemodynamically stable patients with high-grade blunt renal trauma is safe with a low risk of complications. Management decisions should consider hemodynamic status and visualization of active renal bleeding as well as injury grade in determining operative management.

## 1. Introduction

Renal trauma accounts for a significant number of injuries in trauma patients. Up to 10% of patients with abdominal trauma have renal trauma and greater than 90% of those are due to blunt injuries. Approximately 25% of blunt renal injuries are high-grade injuries, meaning grade IV or grade V injuries [1, 2]. Historically, high-grade injuries were managed operatively, which leads to nephrectomy in most cases. However, advancements in resuscitation techniques and interventional radiology have now made the prospect of conservatively managing blunt renal injuries possible. Conservative management is now increasingly preferred in order to avoid nephrectomy and prevent the long-term complications of renal insufficiency [3]. Nonoperative management (NOM) of grade I–III injuries is widely accepted; however management of grade IV and V injuries remains more controversial. Multiple recent studies have investigated the risk factors for and outcome of operative versus NOM in grade IV injuries and the literature increasingly supports conservative management. Grade V injuries, however, are still treated operatively at many institutions and several studies have concluded that grade V injury is a predictor for operative management [4–6]. The most recent literature does include multiple studies suggesting that high-grade injuries, including grade V injuries, can be successfully managed conservatively [3, 7–9].

Our level 1 trauma center has extensive experience in treating renal trauma and currently supports NOM when possible. We hypothesize that NOM is successful in nearly all hemodynamically stable patients with grade IV and V blunt renal injuries. Furthermore, we do not view injury grade as a fixed predictor of need for operation.

## 2. Materials and Methods

Approval was obtained from the Institutional Review Board. We retrospectively reviewed all renal trauma records between October 1, 2004, and December 31, 2013, at Saint Louis University Hospital, a level 1 trauma center. The American Association for the Surgery of Trauma (AAST) grading system was used to define the renal injury grade. Injuries were graded by CT imaging, read by experienced radiologists. A total of 394 blunt renal injuries occurred during this time period, 47 of which were high-grade injuries (grade IV or V). Only patients with high-grade injuries were included in the study. Of these, 39 were of grade IV and 8 were of grade V.

We recorded patient age, gender, injury mechanism, injury severity score (ISS), systolic blood pressure (SBP) on arrival, heart rate (HR) on arrival, hematocrit (Hct) on arrival, hemoglobin (Hg) on arrival, Glasgow Coma Scale (GCS) score, and the presence or absence of liver, splenic, or other abdominal injuries. We also recorded whether free abdominal blood, active extravasation, or urinary extravasation was present on imaging. We recorded any preexisting kidney-related morbidities (renal cyst, horseshoe kidney, hypertension, chronic renal insufficiency, and renal calculi) and non-kidney-related morbidities (asthma, GERD, diabetes, and depression being the most common).

The primary outcome of the study was the type of initial management, including immediate operation (IO) or nonoperative management (NOM). IO was defined as any case requiring immediate transfer to the operative room for exploratory laparotomy in which the renal fascia was opened. Patients that underwent laparotomy without opening of the renal fascia were considered NOM. Patients who underwent stent placement or angioembolization were considered NOM. Other outcomes investigated were complications (including urinoma, hematuria, local abscess, UTI, renal failure, wound infection, DVT, pneumonia, need for subsequent surgeries, and other general complications), and mortality.

In general, all hemodynamically stable patients were considered as candidates for conservative management regardless of injury grade, in accordance with AUA urologic trauma guidelines which state that noninvasive management should be used in hemodynamically stable patients [10]. Hemodynamically stable patients with continued blood loss underwent treatment with embolization or laparotomy. Conservative management included bed rest, serial hemoglobin testing, analgesia, and fluid replacement as needed.

Data was collected from patients' electronic medical records and paper charts. The variables listed above were used to determine risk factors for the need for IO. Univariate analysis was done using Fisher's test for categorical data and Student's *t*-test for continuous data. The same method was repeated for the outcome variables to determine any difference in short-term outcome between the IO and NOM groups. Data was statistically significant with a *P* value of less than 0.05.

## 3. Results

We identified 47 high-grade blunt renal injuries from October 1, 2004, to December 31, 2013. Of the patients included in the study, 33 (70%) were male. The mean age was 36.9 years. The mechanism of injury was most commonly motor vehicle accidents, present in 40 of the 47 patients. Other causes of injury included fall (4 patients), assault (2 patients), and suicide/self-injury (1 patient). Isolated kidney injury was present in 12 while 35 had additional abdominal trauma. Three patients were treated with immediate operation resulting in nephrectomy. Decision for IO was based on visualization of active bleeding from kidney or renal vasculature for two patients, both of whom were stable initially for CT scan. CT scan showed active bleeding from the renal vasculature and both patients were brought to the OR when vital signs became increasingly unstable within the first 24 hours of arrival to the hospital. For both patients, pulsatile or expanding hematoma was noted intraoperatively, necessitating the need to enter the renal fascia. The third IO patient was stable on arrival and CT showed renal artery disruption and zero opacification of kidney with contrast. This patient was brought to the OR immediately after CT scan. A large retroperitoneal hematoma was noted intraoperatively; however there was no indication that the hematoma was expanding or pulsatile. It is presumed that the kidney was removed due to complete disruption of the artery and to prevent later need for nephrectomy as there was zero opacification on CT. This last patient may be a patient that could have been a candidate for initial NOM but would likely require nephrectomy at a later point.

The average age was 34 in the IO group and 37 in the NOM group with no statistical difference. The patient gender and mechanism of injury were not significantly different between the groups. Heart rate on admission was higher in the IO group at 133 compared to the NOM group at 100 (*P* = 0.01). Concomitant liver injury was a significant predictor for IO and was present in all 3 patients in the IO group and 13 patients in the NOM group (*P* = 0.035). Systolic blood pressure (SBP) and injury severity score (ISS) were higher in the IO group and Hct and Hg were lower in the IO group; however none of these measures were found to be statistically significant. Glasgow Coma Scale (GCS) and presence of spleen injury or other abdominal injury were not found to be significantly different between the groups. The IO group included 1 grade IV injury and 2 grade V injuries while the NOM group included 38 grade IV injuries and 6 grade V injuries. We found that injury grade was not a statistically significant risk factor for operative management (*P* = 0.07). Free abdominal blood, whether diffuse or confined, was not different between the groups and neither was presence or absence of active extravasation or urinary extravasation (Table 1).

Due to the nature of the trauma patient population, long-term follow-up was not possible. In the NOM group, short-term complications included one patient with UTI, hematuria, and right flank abscess, three patients with UTI, four patients requiring need for angioembolization, and one patient requiring need for stent placement. There were no short-term complications in the IO group other than mortality. Total complications were not significantly different between the two groups. Difference in mortality was statistically significant with 2 deaths (67%) in the IO group and two deaths (4.5%) in the NOM group. One death in the IO group was due to hypoxia not thought to be related to the kidney injury and the other death was due to ventricular fibrillation in a patient with multiple injuries including chest, pancreas, liver, and splenic injuries. The two deaths in the NOM group were due to withdrawal of care after lack of improvement of neurologic function, not related to kidney injury (Table 2).

The renal salvage rate for the entire cohort was 87.2% and 93.2% for patients managed nonoperatively. NOM failed in 3 of the 44 patients (6.8%) who all required nephrectomy at a later date. One patient originally underwent exploratory laparotomy for hemodynamic instability and required splenectomy at that time. In this patient, a retroperitoneal hematoma was noted during exploration but appeared stable so renal fascia was not entered. After return to ICU the patient became hemodynamically unstable and was returned to the operating room at which time the renal fascia was entered while attempting to isolate the kidney and required emergent nephrectomy. The patient later expired due to hypoxia. This patient also had a concurrent significant head injury that likely contributed to the outcome. The other two patients with failed NOM required nonemergent nephrectomy at a later date for nonfunctioning kidney, one secondary to the original renal trauma and one confounded by congenital UPJ obstruction.

## 4. Discussion

Decision-making in high-grade blunt renal injuries is difficult due to conflicting recommendations in the literature. In addition, renal trauma is rarely an isolated event and often occurs in the presence of multiple other injuries. Thus, the surgeon must use multiple clinical and radiologic factors to determine the proper course of treatment.

While it is universally agreed upon that life-threatening hemorrhage requires immediate operation, less clear-cut indications are still heavily debated. There are multiple recent studies in the literature debating the indications for operation in high-grade injuries. Hardee et al. retrospectively reviewed 115 charts of patients with grade 3 or 4 blunt renal injuries. The intervention rate was 7% with nephrectomy being the most common procedure. Active vascular extravasation and perinephric hematoma > 3.5 cm were predictive of operative treatment [11]. Buckley and McAninch retrospectively reviewed 153 patients with grade 4 renal injury (66 patients with blunt mechanism of injury) of which 103 underwent operative management (26 with blunt injury). Hemodynamic instability and persistent bleeding were causes for IO [12].

Many institutions have found the presence of a grade V injury to be a predictor for the need of operative treatment. Aragona et al. retrospectively reviewed 45 patients with high-grade blunt renal trauma, 6 of which have grade V injuries. The study concluded that conservative management yielded favorable results; however over 80% of grade V injuries required operation [13]. McGuire et al. studied 117 patients with grade 3–5 blunt renal injuries and found that clinical factors indicating need for operative management were need for platelet transfusion and grade V injury [4].

Other studies support the success of conservative management for grade V injuries in hemodynamically stable patients. Altman et al. studied 13 patients with grade V renal injury, 6 of which underwent nonoperative management. They found that patients with nonoperative management had fewer days in ICU, less transfusion requirements, and fewer complications. They concluded, as we have, that grade V injuries can be successfully managed nonoperatively if the patient is hemodynamically stable [7].

Our findings validate the findings of many recent studies suggesting that a trend towards conservative management in high-grade blunt renal trauma is warranted. Of all factors studied, only heart rate at admission and concomitant liver injury were predictive of need for immediate operative intervention. Heart rate may have been the clearest measurement that differentiated hemodynamic stability or instability in our patient cohort. However, it is also possible that elevated heart rate may not have been a result of hypotension for blood loss alone. Pain and anxiety can also result in an elevated heart rate.

Perhaps the most interesting finding in our study compared to the majority of the literature was that grade IV versus grade V injury was not a risk factor for operative management. This is in disagreement with some of the current literature and is an important finding because it suggests that perhaps decisions should be made based on grade of injury in conjunction with hemodynamic status rather than solely on grade of injury.

There are recognized limitations in this study. This was a retrospective study with a cohort of 47 patients and the data from this study may not be applicable to the wider population. There were also multiple confounding factors that complicate the data. This is in large part due to the nature of a trauma population as patients are likely to have other concurrent injuries that can impact the decision to operate or not. Multiple patients in our study were brought to the operating room for concomitant injuries requiring splenectomy or other interventions, but not requiring entry of the renal fascia. In these NOM patients, if a retroperitoneal hematoma was noted but found to be stable and nonexpanding, the renal fascia was left intact in order to allow the hematoma to tamponade itself in the retroperitoneum. In one IO patient, a nonexpanding retroperitoneal hematoma was found on exploration and the decision was made to enter the renal fascia, resulting in nephrectomy. It is possible that this patient could have been managed nonoperatively. Due to the retrospective nature of the study, we were unable to account for differences in management decisions among trauma surgeons. In addition, we were unable to account for the likelihood of concomitant injuries contributing to mortality in both the IO and NOM group. The patients who were operatively managed had less stable vital signs and may have had more serious concurrent injuries, which could have contributed to the higher mortality in the IO group than the NOM group. However, we were unable to characterize the severity of concomitant injuries in this study.

Another recognized potential limitation of the study is that the grade of injury was taken from radiologic report, which can vary between individual radiologists. We were also unable to obtain data regarding the timing of the initial injury and the length of time elapsing until operative intervention or death. In addition, short-term outcomes such as the ones we studied are not adequate to assess success of management; however the nature of the patient population makes longer follow-up difficult as many patients were brought in for their injury and had follow-up elsewhere.

Given the relatively small number of high-grade blunt renal injuries presenting to even large trauma centers annually and the even smaller number of specifically grade V injuries, there is need for more data to determine indications and success of conservative management. Therefore, it is necessary to validate the outcome of NOM at multiple institutions. A meta-analysis of these studies would be beneficial to determine clear-cut criteria for high-grade blunt renal trauma management.

## 5. Conclusion

Nonoperative management of high-grade blunt renal trauma is successful in most hemodynamically stable patients, regardless of injury grade. The rate of failure in NOM patients is very low. In our study, heart rate was the most predictive determinant of hemodynamic stability or instability. We found that heart rate and concomitant liver injury were predictive of immediate operative management. Our findings suggest that a system based on factors measuring hemodynamic status, in addition to injury grade, should play the defining role when determining whether or not to immediately operate on a patient with blunt renal injury.

## Figures and Tables

**Table 1:** Preoperative characteristics in IO versus NOM patients.

Characteristic	IO patients (*n* = 3)	NOM patients (*n* = 44)	*P* value
ISS, mean (SD)	30.3 (10.8)	27.3 (14.8)	0.73
SBP on adm, mean (SD), mm Hg	118 (42)	124 (27)	0.72
HR on adm, mean (SD), beats/min	133 (25)	100 (22)	0.015
HCT on adm, mean (SD), %	32.8 (8.9)	36.5 (5.7)	0.30
Hg on adm, mean (SD), g/dL	10.9 (3.1)	12.6 (1.9)	0.16
GCS score, mean (SD)	11.0 (6.9)	12.9 (4.2)	0.47
Liver injury, number (%)	3 (100)	13 (30)	0.035
Splenic injury, number (%)	1 (33)	19 (43)	1
Other abdominal injury, number (%)	1 (33)	19 (43)	1
Any concurrent abdominal injury (%)	3 (100)	32 (73)	0.56
CT grade of renal injury, number (%)			
(i) IV	1 (33)	38 (86)	0.071
(ii) V	2 (67)	6 (14)	
Free abd blood on CT, number (%)			
(i) Diffuse	1 (33)	7 (16)	0.44
(ii) Confined	2 (67)	29 (66)	1
(iii) None	0	8 (18)	1
Extravasation, number (%)			
(i) Active	2 (67)	15 (34)	0.54
(ii) Urinary	0	3 (7)	1.00
(iii) None	1 (33)	26 (59)	0.57
Morbidity, number (%)			
(i) Kidney-related	0	6 (14)	1
(ii) Non-kidney-related	0	8 (18)	1
(iii) None	0	30 (68)	1

**Table 2:** Short-term outcomes in IO versus NOM patients.

Outcome	IO patients (*n* = 3)	NOM patients (*n* = 44)	*P* value
Complications, number (%)			
(i) UTI	0	4 (9)	
(ii) Abscess, hematuria, UTI	0	1 (2)	
(iii) Bacteremia	0	1 (2)	
(iv) Angioembolization	0	4 (9)	
(v) Stent placement	0	1 (2)	
Total	0	11 (25)	
Mortality, number (%)	2 (67)	2 (5)	0.016

## References

[1] Moore E. E., Shackford S. R., Pachter H. L. (1989). Organ injury scaling: spleen, liver, and kidney. *Journal of Trauma*.

[2] Prasad N. H., Devraj R., Chandriah G. R., Sagar S. V., Reddy C. R., Murthy P. V. L. N. (2014). Predictors of nephrectomy in high grade blunt renal trauma patients treated primarily with conservative intent. *Indian Journal of Urology*.

[3] Shoobridge J. J., Corcoran N. M., Martin K. A., Koukounaras J., Royce P. J., Bultitude M. F. (2011). Contemporary management of renal trauma. *Reviews in Urology*.

[4] McGuire J., Bultitude M. F., Davis P., Koukounaras J., Royce P. L., Corcoran N. M. (2011). Predictors of outcome for blunt high grade renal injury treated with conservative intent. *Journal of Urology*.

[5] Wright J. L., Nathens A. B., Rivara F. P., Wessells H. (2006). Renal and extrarenal predictors of nephrectomy from the National Trauma Data Bank. *Journal of Urology*.

[6] Shariat S. F., Roehrborn C. G., Karakiewicz P. I., Dhami G., Stage K. H. (2007). Evidence-based validation of the predictive value of the American Association for the Surgery of Trauma kidney injury scale. *Journal of Trauma—Injury, Infection and Critical Care*.

[7] Altman A. L., Haas C., Dinchman K. H., Spirnak J. P. (2000). Selective nonoperative management of blunt grade 5 renal injury. *The Journal of Urology*.

[8] Broghammer J. A., Fisher M. B., Santucci R. A. (2007). Conservative management of renal trauma: a review. *Urology*.

[9] Bozeman C., Carver B., Zabari G., Caldito G., Venable D. (2004). Selective operative management of major blunt renal trauma. *Journal of Trauma*.

[10] Morey A. F., Brandes S., Dugi D. D. (2014). Urotrauma: AUA guideline. *The Journal of Urology*.

[11] Hardee M. J., Lowrance W., Brant W. O., Presson A. P., Stevens M. H., Myers J. B. (2013). High grade renal injuries: application of Parkland Hospital predictors of intervention for renal hemorrhage. *The Journal of Urology*.

[12] Buckley J. C., McAninch J. W. (2006). Selective management of isolated and nonisolated grade IV renal injuries. *Journal of Urology*.

[13] Aragona F., Pepe P., Patanã D., Malfa P., D'Arrigo L., Pennisi M. (2012). Management of severe blunt renal trauma in adult patients: a 10-year retrospective review from an emergency hospital. *BJU International*.

